# HPiP: an R/Bioconductor package for predicting host–pathogen protein–protein interactions from protein sequences using ensemble machine learning approach

**DOI:** 10.1093/bioadv/vbac038

**Published:** 2022-05-23

**Authors:** Matineh Rahmatbakhsh, Mohamed Taha Moutaoufik, Alla Gagarinova, Mohan Babu

**Affiliations:** 1 Department of Biochemistry, University of Regina, Regina, SK S4S 0A2, Canada; 2 Department of Biochemistry, University of Saskatchewan, Saskatoon, SK S7N 5E5, Canada

## Abstract

**Motivation:**

Despite arduous and time-consuming experimental efforts, protein–protein interactions (PPIs) for many pathogenic microbes with their human host are still unknown, limiting our understanding of the intricate interactions during infection and the identification of therapeutic targets. Since computational tools offer a promising alternative, we developed an R/Bioconductor package, HPiP (Host–Pathogen Interaction Prediction) software with a series of amino acid sequence property descriptors and an ensemble machine learning classifiers to predict the yet unmapped interactions between pathogen and host proteins.

**Results:**

Using severe acute respiratory syndrome coronavirus 1 (SARS-CoV-1) or the novel SARS-CoV-2 coronavirus-human PPI training sets as a case study, we show that HPiP achieves a good performance with PPI predictions between SARS-CoV-2 and human proteins, which we confirmed experimentally in human monocyte THP-1 cells, and with several quality control metrics. HPiP also exhibited strong performance in accurately predicting the previously reported PPIs when tested against the sequences of pathogenic bacteria, *Mycobacterium tuberculosis* and human proteins. Collectively, our fully documented HPiP software will hasten the exploration of PPIs for a systems-level understanding of many understudied pathogens and uncover molecular targets for repurposing existing drugs.

**Availability and implementation:**

HPiP is released as an open-source code under the MIT license that is freely available on GitHub (https://github.com/BabuLab-UofR/HPiP) as well as on Bioconductor (http://bioconductor.org/packages/devel/bioc/html/HPiP.html).

**Supplementary information:**

Supplementary data are available at *Bioinformatics Advances* online.

## 1 Introduction

Changes in the patterns of microbial infections, as well as the emergence of multidrug-resistant strains (superbugs) among pathogens, have deteriorated the efficacy of existing therapeutics and limited current treatment options (Larsson and Flach, 2022). This necessitates the perpetual identification and refinement of host–pathogen protein–protein interactions (HP-PPIs), which is vital for understanding the molecular basis of underlying protein functions, interactions that result in infection outcomes and how pathogens infect and subvert host functions by recruiting them for their own cellular activities (Nicod *et al.*, 2017). Given their importance in disease, detecting HP-PPIs, both computationally and experimentally, has gained importance as one of the key contributors for the development of therapeutic interventions to combat infectious diseases (Jean Beltran *et al.*, 2017; Rahmatbakhsh *et al.*, 2021). Performing biochemical and genetic experimental methods to detect PPIs between all proteins of a pathogen and their host is time-consuming and labor-intensive due to their genomic complexity. Even for well-studied pathogens, experimentally derived PPIs between the pathogen and host proteins cover only a fraction of the projected interaction space in the network. This highlights the need for less time-consuming and affordable computational methods such as protein sequence, structure or domain information, coexpression profiles, genome sequences, functional similarity and gene neighborhood to predict undetected and biologically relevant HP-PPIs which can complement experimentally identified datasets to formulate hypotheses. Yet, these PPI descriptors are not always available, limiting their application.

While querying protein pairs using 3D structures is limited by low proteome coverage, computational approaches using protein sequence information are more amenable to investigating PPI networks due to the advancements in next-generation sequencing data of pathogens in recent years. However, there are a few shortcomings. First, only a small selection of physicochemical descriptors such as amino acid composition (i.e. di- or tri-peptide), as well as k-Spaced amino acid pairs and Conjoint Triad have been considered, but combining these with other descriptors can detect *de novo* PPIs based on the primary sequences of proteins. Second, continuously running iterations by sequence-based computational methods can become cumbersome due to sizeable run time required to train a scoring model, and when handling large number of sequences. Third, while HP-PPI predictions using sequence-based methods are available through a few web server platforms, such as PredHPI, they lack programming interfaces and are not designed to analyze a sizeable number of sequences in a single batch. Also, in such web applications, it is often unclear the type of reference interactions used to evaluate HP-PPI predictions, especially when PPIs are unavailable for many host–pathogen systems.

Conversely, progress in machine learning (ML)-based methods using supervised learning algorithms such as Support Vector Machine (SVM), Random Forest (RF) and Logistic Regression (LR) is emerging as an alternate approach to predict PPIs based on physicochemical properties of amino acid sequences, and discern interacting from non-interacting protein pairs using known interactors as a training set. Nevertheless, most studies reported only the results of cross-validation (CV), with no reference to the evaluation of test prediction results on a new set of data using rigorous experimental or external validations. While these issues may challenge the interpretability of prediction performance in deciphering genuine interactions, with an ensemble ML methods and experimental evaluation, prediction accuracy and coverage of PPIs can be enhanced. However, current ML approaches that predict HP-PPIs are not automated, and therefore end-user must obtain suitable training or test datasets, and needs in-depth ML knowledge.

In this study, we address the aforesaid gaps by introducing an R/Bioconductor software package, HPiP (Host–Pathogen Interaction Prediction), built on an ensemble approach with three (SVM, RL and LR) different ML classifiers, and a pipeline to either automatically or manually upload the training dataset, and predict PPIs based on more than a dozen amino acid sequence property descriptors of pathogen and host proteins. As a case study, using SARS-CoV-1 or the SARS-CoV-2-human PPI training set, HPiP achieved a good performance with PPI prediction between SARS-CoV-2 and human proteins using ensemble ML method. Besides verifying PPIs with 10-fold CV, published datasets and quality control metrics, we experimentally confirmed the predicted PPIs in human THP-1 monocytes by affinity purification-mass spectrometry (AP/MS). Similar high performance on the PPI prediction by HPiP was achieved when tested against the bacterial pathogen, *Mycobacterium tuberculosis* and human protein sequences. Source code, standalone executable programs, usage instructions, illustrative dataset and output for testing are provided at https://github.com/BabuLab-UofR/HPiP and in Supplementary Tables S1–S8.

## 2 HPiP R package software environment

**Fig. 1.**
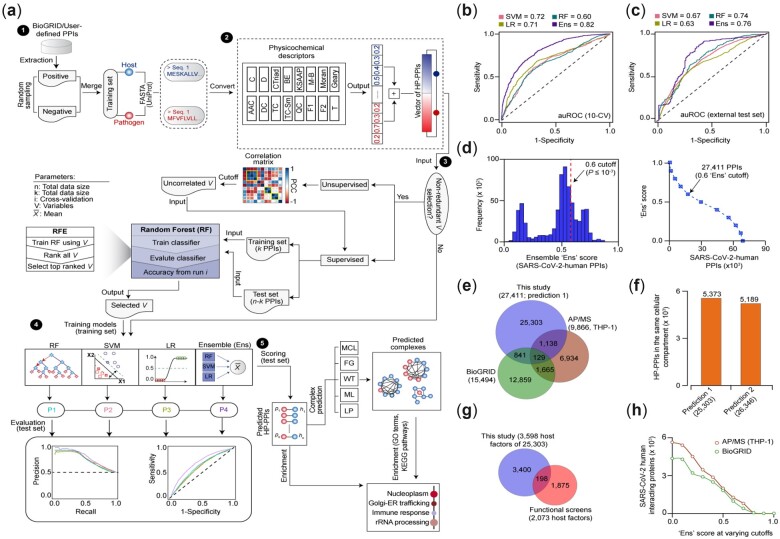
HPiP workflow, parameter evaluation and validation of predicted interactions. (**a**) Five-step computational pipeline to predict HP-PPIs: (i) Building literature-curated training set; (ii) Converting amino acid sequences to numerical values using physicochemical descriptors; (iii) Selecting protein sequence-based numerical variables from different descriptors as input for ML; (iv) Fine-tuning ML model parameters using the training set, followed by CV and model performance evaluation; as well as (v) Scoring HP-PPIs in test set, and predicting multiprotein complexes from HP-PPI network using fast greedy (FG), walk-trap (WT), label propagation (LP), multilevel community (ML) and Markov clustering (MCL) methods. Putative interacting protein pairs were subjected to functional enrichment analysis for associations to biological functions using GO and KEGG pathway terms. For acronyms on physicochemical descriptors, see Supplementary Table S1. RFE, recursive feature elimination; SVM, support vector machine; LR, logistic regression. (**b, c**) auROC showing the performance measures of ensemble versus other ML classifiers evaluated using 10-fold CV in a training (b) and test (c) sets. (**d**) Histogram (left) and distribution (right) of SARS-CoV-2–human protein interaction pairs at various ensemble score; arrow with ensemble cutoff score ≥0.6 imply statistically significant (*P*-value ≤ 10^−3^) interactions; *P*-value computed by permutation test. (**e**) Overlap of SARS-CoV-2-human PPIs predicted from this study versus literature-curated interactions from BioGRID database and experimentally derived PPIs from THP-1 cells using AP/MS method. (**f, g**) Evidence supporting sequence-based prediction of SARS-CoV-2-human PPIs by colocalization to same cellular compartment (f) criteria, and human host factors interacting with SARS-CoV-2 proteins that were vital for coronavirus infections based on published genetic screens (g). The number in parenthesis indicates total number of interactions (e, f) or host factors (g) used for comparison. (**h**) Overlap of sequence-based prediction of SARS-CoV-2-human PPIs versus associations detected by AP/MS in THP-1 cells or in the BioGRID database at varying ensemble score cutoffs

The HPiP software is composed of a five-step automated computational workflow (Fig. 1a) to predict HP-PPIs. In Step 1, the curated HP-PPIs were collected from BioGRID database of an organism under study. These curated host–pathogen interaction protein pairs were considered for training as positives, and non-interacting pairs as negatives, displaying distinct distributions as expected (Supplementary Fig. S1a). Alternately, HPiP allows users to submit their own curated reference dataset to optimize training. In Step 2, the amino acid sequences for pathogen and host proteins were converted into numerical variables from each of the 16 selected physicochemical descriptors (Supplementary Table S1), and the resulting values of two individual proteins (i.e. host and pathogen) were concatenated. In Step 3, HPiP combines unsupervised (i.e. correlation-based) and supervised (i.e. RF-based recursive feature elimination) methods to filter redundant numerical variables based on the user-defined threshold of Pearson correlation coefficient and weights given by RF to each variable, respectively. In Step 4, three ML classifiers (i.e. SVM, RF and LR) were independently and collectively assessed with the training dataset to fine-tune model parameters and comparative performance evaluation using *k*-fold CV, but other ML classifiers can be substituted with parameters supported by the caret package (https://github.com/topepo/caret/). The best parameters are defined based on the model prediction, while a model’s predictive performance was evaluated using precision versus recall, or sensitivity versus 1-specificity plots. In the final Step 5, either individual or ensemble (i.e. average of predictions) of tuned ML classifiers is subjected to the test set for scoring putative HP-PPIs. The resulting interactions are split into complex membership using fast greedy, walk-trap, label propagation, multilevel community and Markov clustering algorithms that are available in HPiP package, though users can define complexes using other clustering methods such as ClusterONE via an open-source Cytoscape platform. HPiP then uses scored PPIs or predicted complexes to perform functional enrichment using Gene Ontology (GO; i.e. molecular function, cellular component and biological processes) or KEGG (Kyoto Encyclopedia of Genes and Genomes) pathway terms to define bioprocesses involving host-dependency factor for pathogens.

## 3 Study cases

To illustrate our approach, we optimized HPiP performance to predict SARS-CoV-2-human host PPIs in two ways. In the first method (referred as ‘prediction 1’), 1562 interactions were gathered for training from the closely related SARS-CoV-1 coronavirus with human host proteins (Gordon *et al.*, 2020). In the second method (referred as ‘prediction 2’), 4648 SARS-CoV-2-human PPIs from the BioGRID database were compiled, and we randomly withheld 70% of these PPIs for training, while the remaining 30% for testing. In prediction 1 method, we considered 1562 interactions measured using MiST (MS interaction statistics) algorithm with a scoring threshold greater than 0.5 (Gordon *et al.*, 2020) as a positive training set. For negative set, we generated 40 124 non-interacting pairs from a random pairing of human and SARS-CoV-1 proteins in the positive dataset. To avoid prediction bias, we chose an equal number (i.e. 1562) of positive and negative PPI pairs for training (Supplementary Table S2).

Evaluation of sequence descriptors by 10-fold CV in training showed a robust performance of 0.76 area under the receiver operating characteristic curve (auROC) can be achieved when all the descriptors were combined compared to 0.75 average training accuracy for each descriptor (Supplementary Fig. S1b). Assessment of three ML classifiers, individually and collectively, against our training dataset showed that ensemble classifier outperformed with a highest composite score (auROC = 0.82; Fig. 1b) than the individual ML model (average auROC = 0.70) for predicting HP-PPIs. Similar performance was attained against the test dataset [i.e. amino acid sequences retrieved from 242 experimentally derived known SARS-CoV-2-human PPIs (Gordon *et al.*, 2020)] (Supplementary Table S3), where ensemble classifier achieved the best performance (auROC = 0.76; Fig. 1c) by capturing 75% (182 of 242) of test interactions. This suggests that by using physical interactions identified in a pathogen that share similar protein sequences in other species with unmapped interactions, our ensemble classifier can provide higher PPI prediction accuracy.

Next, we examined whether our ensemble classifier can predict new human protein associations with SARS-CoV-2 proteins that were previously undetected by experimental approaches. To do so, we followed Steps 2–5 of the HPiP workflow and chose an ensemble classifier cutoff score ≥0.6 (Fig. 1d), which is significant (*P*-value ≤ 10^−3^) by random sampling. These filtered associations tend to decrease gradually as ensemble classifier score was elevated (Fig. 1d), suggesting interactions passing the chosen threshold might be reliable with adequate specificity. In total, the selected score threshold led us to predict 27 411 high-confidence HP-PPIs among 3599 humans and 15 SARS-CoV-2 proteins (Supplementary Table S4). Nearly one-tenth (∼8%, 2108 of 27 411) of these PPIs were confirmed by several independent experimental assessments (Supplementary Table S4). This includes: (i) 1138 host factors interacting with SARS-CoV-2 protein in THP-1 monocytes by AP/MS (Supplementary Methods), (ii) 841 host protein interactions with SARS-CoV-2 proteins were captured in BioGRID based on AP/MS and BioID/MS (i.e. proximity-dependent biotinylation-MS) and (iii) 129 were detected in both BioGRID and AP/MS datasets (Fig. 1e). Confirmed high-confidence protein interaction pairs by AP/MS in THP-1 include SARS-CoV-2 non-structural (NSP2/5/12), structural (nucleocapsid, N) and accessory (ORF3A) proteins with Ras superfamily of small GTPases (Supplementary Fig. S1c) such as Rabs (RAB1B, 4A, 8B, 11B, 35) and class II ADP-ribosylation (ARF4, 5) human factors that are crucial for virus replication, including SARS-CoV-2 (Daniloski *et al.*, 2021; Wang *et al.*, 2021). Also, SARS-CoV-2 proteins bridged human mitochondrial proteins (TIMM13, COX5A, NDUFV1; Supplementary Fig. S1c), implying the virus may evade mitochondrial-mediated immune response to proliferate (Burtscher *et al.*, 2020).

One-fifth (21%; 5373 of 25 303) of the remaining interacting proteins colocalized to the same cellular compartment (Fig. 1f), while 198 human host factors (Fig. 1g) that were physically interacting with SARS-CoV-2 were vital for infections by SARS-CoV-2 or common cold coronaviruses (Daniloski *et al.*, 2021; Gordon *et al.*, 2020; Wang *et al.*, 2021). However, the reliability of the left-over predicted interactions increased as Ensemble score was decreased, indicating these faithfully recapitulated associations failed to pass through because of the set stringent score threshold (Fig. 1h). Assessment on the 27 411 putative SARS-CoV-2-human protein assemblies further revealed significant (Q-value ≤ 0.01) enrichment for a number of cellular process or pathways important for SARS-CoV-2 (Supplementary Fig. S1d and Table S5). This includes neutrophil activation, autophagy, inflammatory immune responses, NF-κB signaling and innate immunity, among others, suggesting that our prediction 1 strategy provides candidate host factors for ensuing validation or host-direction inhibition of viral infection.

Given the possibility that interspecies protein interactions for model training may not be optimal for less conserved understudied pathogens, we employed our prediction 2 approach by making use of the aforesaid known SARS-CoV-2-human PPIs for training and the holdout set for testing (Supplementary Tables S6 and S7) to evaluate ML classifiers. In contrast to other models, the ensemble classifier performed well with our training set based on 10-fold CV (auROC = 0.71, Supplementary Fig. S1e) with 78% (703 of 903; auROC = 0.75; Supplementary Fig. S1f) accuracy to correctly capture the test interactions. Using a similar cutoff strategy as in prediction 1 method (Fig. 1d), we found one-tenth (30%, 8937 of 30 094) of the newly predicted SARS-CoV-2-human protein associations that have not been reported previously was supported by our AP/MS method in THP-1 cells, and/or reported in BioGRID database **(**Supplementary Fig. S1g and Table S8), and colocalize to the same compartment (Fig. 1f). However, as with prediction 1 method, the number of interactions from prediction 2 markedly increased below the stringent threshold, but agreeing favorably with AP/MS and/or BioGRID datasets (Supplementary Fig. S1h). Conversely, we found poor overlap (6.3%, 12 of 192) of PPIs predicted from PredHPI tool against THP-1 cells by AP/MS, and/or reported associations from BioGRID database (Supplementary Fig. S2a), emphasizing the need for developing new software toolkit such as HPiP to predict HP-PPIs with improved performance. In fact, this was the case, where HPiP maximized the coverage and accuracy for half of the interactions (52%; 17 879 of 34 497) from both prediction methods and/or experimental data (Supplementary Fig. S1i and j), underscoring the reproducibility for native physical associations. The remaining high-confidence SARS-CoV-2-human protein assemblies not detected in other studies represent a rich source of potentially undiscovered PPIs to spur further exploration and for gaining new mechanistic insights.

Since SARS-CoV-1 is related to SARS-CoV-2, we inquired whether HPiP can uncover human host protein interactions with low sequence similarity between these coronaviruses. We thus examined the similarity between sequences of the trained SARS-COV-1 and the predicted SARS-COV-2 interacting human host proteins using a pairwise sequence alignment. Strikingly, HPiP detected the human interacting proteins at both high and low sequence similarity (i.e. Supplementary Fig. S2b). This suggests that HP-PPIs can be predicted by HPiP even for pairs of genomes belonging to phylogenetically distant genera with low sequence identity.

Lastly, to showcase that HPiP can predict PPIs for other pathogens at similar precision and accuracy as coronaviruses, we compiled 1118 *M.tuberculosis*-human PPIs involving 45 secreted *M.tuberculosis* proteins using MiST score ≥0.5 (Penn *et al.*, 2018). As with our prediction methods for SARS-CoV-2-human PPIs, for training, we withheld randomly 70% (767 of 1118; 390 as positive and 377 as negative set) of the PPIs, while the other 30% (351 of 1118; 161 as positive and 190 as negative) for testing. In comparison to other ML classifiers, ensemble classifier performed well with the training set based on 10-fold CV (auROC = 0.78) and with 83% (135 of 161; auROC = 0.82) accuracy to capture the test PPIs (Supplementary Fig. S2c and d) akin to other metrics used to measure the performance of ensemble classifier (Supplementary Fig. S2e and f).

## 4 Conclusion

We have developed an open-source HPiP software to facilitate prediction of PPIs from the sequences of host and pathogen proteins using several amino acid sequence descriptors and an ensemble ML classifier to address the current gap of the unmapped physical interactions that remains limited for many understudied or emerging pathogens. HPiP is interfaced with an optimized computational workflow and does not require programming skills to run the automated data analysis pipeline. While HPiP allows users to train ML models using their own curated dataset, as a case study, we show that using known SARS-CoV-1 (prediction 1) or SARS-CoV-2 (prediction 2)*-*human protein interaction training set, an ensemble learning approach can predict previously unreported SARS-CoV-2-human PPIs with high accuracy from protein sequences, which we confirmed by orthogonal methods. Yet, HPiP performance depends largely on the sequence similarity between host and pathogens, and thus the quality of experimental training dataset is vital to train ML classifier. In summary, we expect HPiP to interrogate changes in HP-PPIs which is vital for understanding complex infectious diseases, as well as prompt new testable hypotheses about the function of interacting proteins and their role in disease mechanisms. Extending HPiP to residue–residue coevolution (Cong *et al.*, 2019) across host–pathogen protein interfaces to predict HP-PPIs can further be beneficial to build structural models of protein interactions, and uncover new HP-PPIs that existing computational methods might fail to predict.

## Supplementary Material

vbac038_Supplementary_Data
