# Supplementary material for: HPiP: an R/Bioconductor package for predicting host–pathogen protein–protein interactions from protein sequences using ensemble machine learning approach
Source: Bioinform Adv. 2022 May 23;2(1):vbac038. doi: 10.1093/bioadv/vbac038 (PMC9154073; doi:10.1093/bioadv/vbac038)
Supplement: vbac038_Supplementary_Data [file vbac038_supplementary_data.zip › Supplementary material-May 16-2022.pdf]

SUPPLEMENTARY MATERIAL

1. SUPPLEMENTARY METHODS

1.1. Expression plasmids: The lentiviral expression plasmid (pLVX-EF1alpha) expressing independently each of the 26 SARS-CoV-2 proteins [i.e., 4 structural: spike (S), nucleocapsid (N), membrane (M), envelope (E); 13 non-structural: NSP1, 2, 4, 5, 7-15; and 9 accessory ORFs: ORF3A, 3B, 6, 7A, 7B, 8, 9B, 9C, 10], and GFP vector control with twin-Strep-tag (2x Strep-tag II) was provided by Dr. Krogan (Gordon, et al., 2020).

1.2. Cell culture: Human monocytic THP-1 cells were grown in Roswell Park Memorial Institute (RPMI)-1640 growth medium (Gibco), supplemented with 10 % fetal bovine serum (Gibco) and 1% penicillin-streptomycin (VWR), and maintained in a humidified 5% CO_2 atmosphere at 37°C. The cultured cells were maintained at a density of 10 x 10^6 cells/ml.

1.3. Transfection: Ten million THP-1 cells plated in 4 x 100 mm tissue-culture dishes was transfected with 5 µg of each SARS-CoV-2 expression plasmids carrying 2x Strep-tag II with Lipofectamine LTX reagent (Invitrogen) for 48 hrs. Cells were detached by gentle scraping at room temperature with 4 ml phosphate-buffered saline (PBS), followed by washing cells twice in 10 ml ice-cold PBS. Cells were subsequently cross-linked as we described (Malty, et al., 2017; Moutaoufik, et al., 2019) using membrane-permeable crosslinking reagent dithiobis succinimidyl propionate (0.5 mM, Thermo Fisher), and incubated for 30 min at room temperature. The crosslinking reaction was quenched by treating the THP-1 cells with 100 mM Tris-HCl (pH 7.5) at room temperature for 10 min. Cells collected by gentle pipetting were washed twice with PBS and subjected to centrifugation at 1,300 *xg* for 5 min. Cell pelletes were frozen on dry ice and stored at -80°C prior to use for AP/MS.

1.4. Affinity purification (AP): Frozen cell pellets were thawed and re-suspended in 1 ml lysis buffer (50 mM Tris-HCl, pH 7.4 at 4°C, 150 mM NaCl, 1 mM EDTA) containing 0.5% Nonidet P-40 (NP40; Fluka Analytical) and phosphatase inhibitor cocktail (Sigma-Aldrich) for all SARS-CoV-2 bait proteins, except for spike, where 1% n-Dodecyl-B-D-Maltoside was used instead of NP-40. After pelleting the debris by centrifuation at 14,000 *xg* for 20 min at 4°C, the lysates were affinity purified using MagStrep 'type3' XT beads (Cedarlane) as previously described (Gordon, et al., 2020). The purified proteins eluted with biotin then proceeded to on-bead digestion. Bead-bound proteins were denatured and reduced at 37°C for 30 min using 2 M urea, 50 mM Tris-HCl (pH 8.0) and 1 mM DTT, followed by alkylation with 10 mM iodo-acetamide in the dark for 40 min. The mixture was digested with trypsin gold (Promega) overnight at room temperature by gently mixing with a rocking shaker. The reaction was stopped by adding 2 µL of acetic acid. After equilbirating C18 ZipTip pipet tips as we described (Malty, et al., 2017), the digested samples were aspirated and dispensed in 60% acetonitrile in 0.1% formic acid, and then air-dried and resuspended in 20 ul of 0.1% formic acid for mass spectrometry (MS). For each SARS-CoV-2 bait, 2 independent replicates were prepared for MS.

1.4. MS data acquisition and analysis: Trypitc peptides injected onto the reversed-phase C18 nano-separation column was separated on an EASY-nano high-performance liquid chromatography 1000 (Proxeon, EASY-nLC 1000) following the gradient as we described (Malty, et al., 2017). The eluted peptides from the EASY-nLC were sprayed into the Orbitrap Elite mass spectrometer (Thermo Fisher Scientific) using positive electrospray ionization at an ion source temperature of 250°C and an ion spray voltage of 2.1 kV. Full-scan MS spectra (m/z 350–2000) were acquired in the Orbitrap Elite at a mass resolution of 60,000 (m/z 400), with additional settings for fragementation implemented as we previously described (Malty, et al., 2017). Raw MS/MS spectra were mapped against a reference human protein sequences (downloaded from the UniProt database on July 2020) and appended with reverse decoy sequences to dertermine the false-discovery rate set to less than 5% for peptide and protein identifications. To increase protein coverage, peptide matches from MSGF+ (Granholm, et al., 2014) evaluated by percolator (The, et al., 2016), SEQUEST (ver. 27-rev.9) search engine matches by STATQUEST (Kislinger, et al., 2003), and precursor ion (MS1) intensity aquired by MaxQuant (ver. 2.1.0) (Cox and Mann, 2008) was employed for protein identifications. Peptide search tolerance for MS1 was set to 4.5 ppm with two missed cleavages, while the MS2 fragment ion tolerance was set to 10 ppm and one missed cleavage. Proteins identified in both replicates from each of the three search engines was independently subjected to protein-protein interaction (PPI) scoring framework with ComPASS-Plus (Sowa, et al., 2009). The resulting SARS-CoV-2-human protein associations and their computed scores for each search algorithm was used as an input for random forest (RF) classifier. For training purposes, the curated and known physical interactions were retrieved from the BioGRID database, while non-interacting pairs was constructed using negative sampling from the positive training set (see main text). Predicted PPIs from each search engine that were selected by the RF machine learning classifier was assigned with a RF score, and were later averaged. However, PPIs were deemed to be of high-quality only when they pass through a chosen ≥ 0.5 baseline threshold score. The computational scoring pipeline pertaining to the AP-MS data in THP-1 cells is available on GitHub (https://github.com/ BabuLab-UofR/MSiP).

2. SUPPLEMENTARY TABLES

Supplementary Table S1 - Sequence-based physicochemical descriptors in HPiP software package.

Supplementary Table S2 - SARS-CoV-1-human PPI training set from Gordon et al., 2020 (PMID:7808408; Related to Fig. 1b).

Supplementary Table S3 - SARS-CoV-2-human PPI testing set from Gordon et al., 2020 (PMID:7808408; Related to Fig. 1c).

Supplementary Table S4 - Prediction 1 method highlighting the validation of high-confidence SARS-CoV-2-human PPIs with experimentally-derived dataset and literature studies using SARS-CoV-1-human PPIs as a training dataset (Related to Figs. 1d-g; Supplementary Fig. S1c).

Supplementary Table S5 - Enrichment of SARS-CoV-2-human interacting factors with shared GO annotations/KEGG pathway terms using SARS-CoV-1 as training set (Related to Supplementary Fig. S1d).

Supplementary Table S6 – 70% of the SARS-CoV-2-human PPI training set from BioGRID database (Related to Supplementary Fig. 1e).

Supplementary Table S7 – 30% of the SARS-CoV-2-human PPI testing set from BioGRID database (Related to Supplementary Fig. 1f).

Supplementary Table S8 – Prediction 2 method highlighting the validation of high-confidence SARS-CoV-2-human PPIs with experimentally-derived dataset and literature studies using SARS-CoV-2-human PPIs as a training dataset (Related to Supplementary Fig. 1g).

3. SUPPLEMENTARY FIGURES

Supplementary Fig. 1 - Parameter evaluation, and validation of putative sequence-based SARS-CoV-2-human interactions based on two prediction strategies. **(A)** Density curves of the positive (known or interacting, brown) and negative (randomized or non-interacting, blue) protein interaction pairs, as well as positive-to-negative ratio (green) are shown with distinct ensemble prediction score distributions. **(B, E, F)** Area under the receiver operating characteristic curve (auROC) displaying the performance measure of each physicochemical descriptors vs. all descriptors combined (B), as well as the performance of ensemble vs. other machine learning classifiers (E, F) using 10-fold cross-validation (CV) in a training (B, E) and test (F) datasets. **(C)** Heatmap portraying the patterns (i.e., based on ensemble prediction score) of human proteins associated with the indicated SARS-CoV-2 viral baits predicted by HPiP sequence-based method confirmed in THP-1 monocyte

cells. White cells correspond to SARS-CoV-2-human interacting proteins detected below the select threshold of ensemble cut-off score ≥ 0.6. **(D**) The indicated gene ontology (GO) (i..e, MF, molecular function; CC, cellular component, and BP, biological pocess) and KEGG pathway terms significantly enriched (*Q*-value ≤ 0.05, shown as circles) for human host factors (i.e., 3,598) interacting with SARS-CoV-2 viral bait proteins. **(G)** Overlap of SARS-CoV-2-human PPIs from this study (based on prediction 2 strategy) compared to interactions from BioGRID and AP/MS experiments in THP-1 cells. **(H)** Overlap of sequence-based prediction of SARS-CoV-2-human PPIs to associations detected by AP/MS experiments in THP-1 cells or in the BioGRID PPI studies at varying ensemble score cut-offs. **(I, J)** Venn diagram (I) shows the overlap of sequence-based prediction of SARS-CoV-2-human PPIs by two different methods compared to experimentally-derived interactions from the BioGRID database and AP/MS experiments in THP-1 cells, as well as evidence supporting the prediction of SARS-CoV-2-human PPIs by two different methods based on their co-localization (J) to same cellular compartment. Number in parenthesis indicate total number of interactions (G,I) used in the supporting evidence for comparison or associations lacking no experimental evidence (J) either from the BioGRID database or AP/MS proteomic screens.

Supplementary Fig. 2 - Evalution of PredHPI, as well as HPiP assessment on predicting human PPIs for a bacterial pathogen. **(A)** Overlap of SARS-CoV-2-human PPIs predicted from PredHPI to literature-curated interactions from BioGRID and experimentally-derived PPIs from the human monocyte THP-1 cells using AP/MS. **(B)** Boxplot showing the distribution of sequence similairty based on pairwise alignment of the human host interacting proteins of SARS-COV-1 from the training set against the predicted SARS-COV-2-human PPIs. **(C, D)** Area under the receiver operating characteristic curve (auROC) showing the performance measures of ensemble ('Ens') vs. other machine learning classifiers evaluated using 10-fold cross-validation (CV) in a training (B) and test (C) sets of the *Mycobacterium tuberculosis* (Mtb)-human PPIs. **(E)** Evaluation of HPiP by the indicated performance metrics with Mtb-human PPI test dataset; PPV, Positive predictive value. **(F)** Comparison of 'Ens' classifier predicted score for Mtb-human PPI test dataset against the corresponding PPI pairs with MiST (MS interaction statistics) scores as previously reported (Penn, et al., 2018).

4. SUPPLEMENTARY DATA

HPiP source code, tutiorial vignettes, usage instructions, and supporting training or test datasets to predict host-pathogen interactions are available on the GitHub (https://github.com/BabuLab-UofR/HPiP) and Bioconductor (http://bioconductor.org/ packages/devel/bioc/html/HPiP.html), as well as in **Supplementary Tables S1-S8**.

5. REFERENCES

Cox, J. and Mann, M. (2008) MaxQuant enables high peptide identification rates, individualized p.p.b.-range mass accuracies and proteome-wide protein quantification. *Nat. Biotechnol.*, 26, 1367-1372.

Gordon, D.E., et al. (2020) A SARS-CoV-2 protein interaction map reveals targets for drug repurposing. *Nature*, 583, 459-468..

Granholm, V., et al. (2014) Fast and accurate database searches with MS-GF+Percolator. *J. Proteome Res.*, 13, 890-897.

Kislinger, T., et al. (2003) PRISM, a generic large scale proteomic investigation strategy for mammals. *Mol. Cell Proteomics,* 2, 96-106.

Malty, R.H., et al. (2017) A Map of Human Mitochondrial Protein Interactions Linked to Neurodegeneration Reveals New Mechanisms of Redox Homeostasis and NF-kappaB Signaling. *Cell Syst.*, 5, 1-14.

Moutaoufik, M.T., et al. (2019) Rewiring of the human mitochondrial interactome during neuronal reprogramming reveals regulators of the respirasome and neurogenesis. *iScience*, 19, 1114-1132.

Penn, B.H., *et al.* (2018) An Mtb-human protein-protein interaction map identifies a switch between host antiviral and antibacterial responses. *Mol Cell,* 71, 637-648.

Sowa, M.E., et al. (2009) Defining the human deubiquitinating enzyme interaction landscape. *Cell*, 138, 389-403.

The, M., et al. (2016) Fast and accurate protein false discovery rates on large-scale proteomics data sets with percolator 3.0. *J. Am. Soc. Mass Spectrom.*, 27, 1719-1727.
