# Supplementary figures and images for: HPiP: an R/Bioconductor package for predicting host–pathogen protein–protein interactions from protein sequences using ensemble machine learning approach
Source: Bioinform Adv. 2022 May 23;2(1):vbac038. doi: 10.1093/bioadv/vbac038 (PMC9154073; doi:10.1093/bioadv/vbac038)

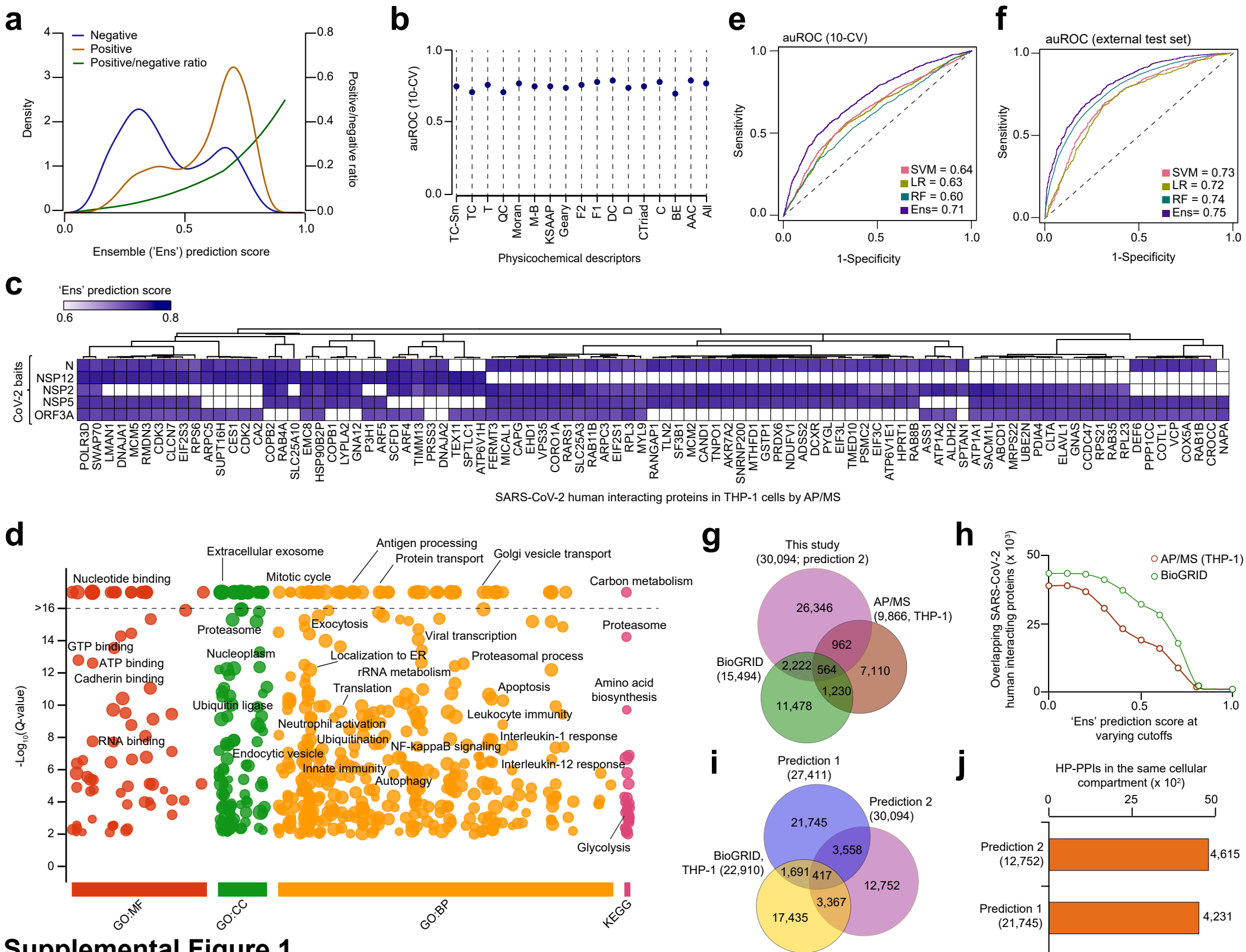

**Supplemental Figure 1**

Supplement: vbac038_Supplementary_Data [file vbac038_supplementary_data.zip › Supplementary_Figure1.pdf]

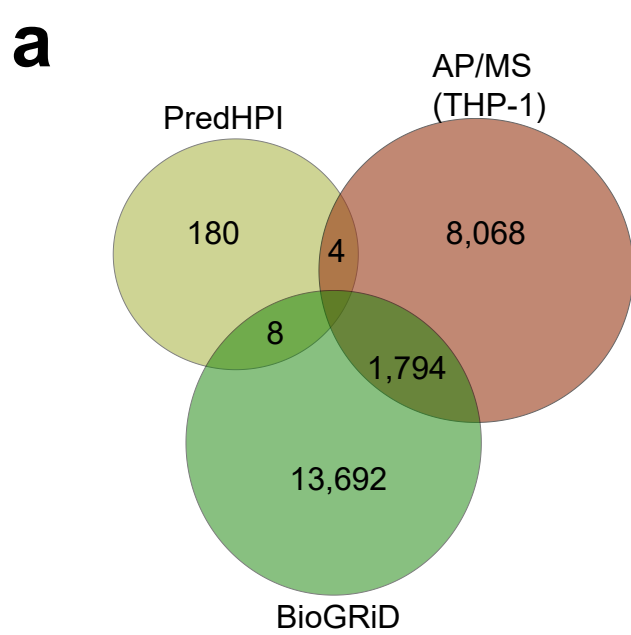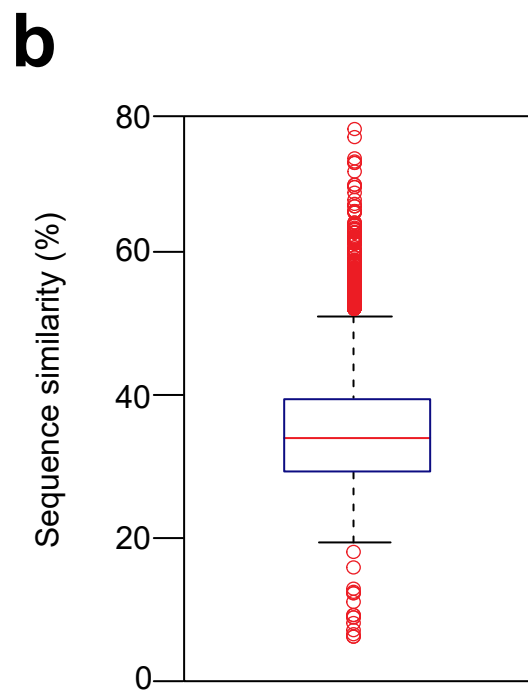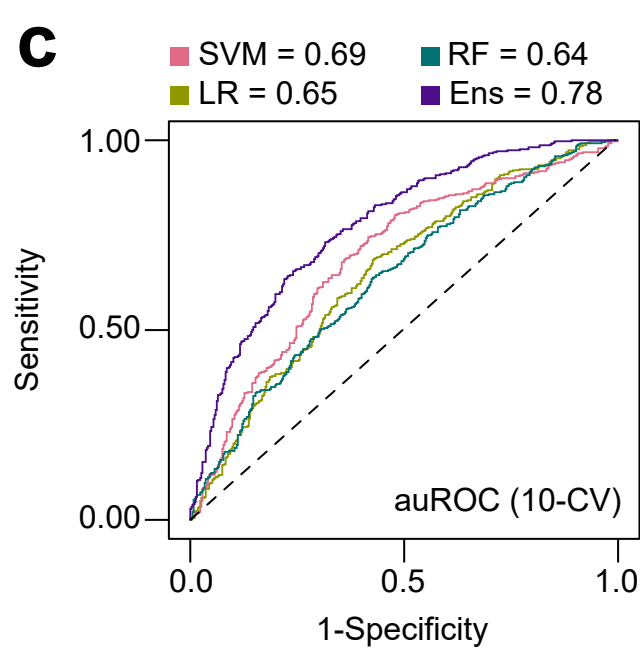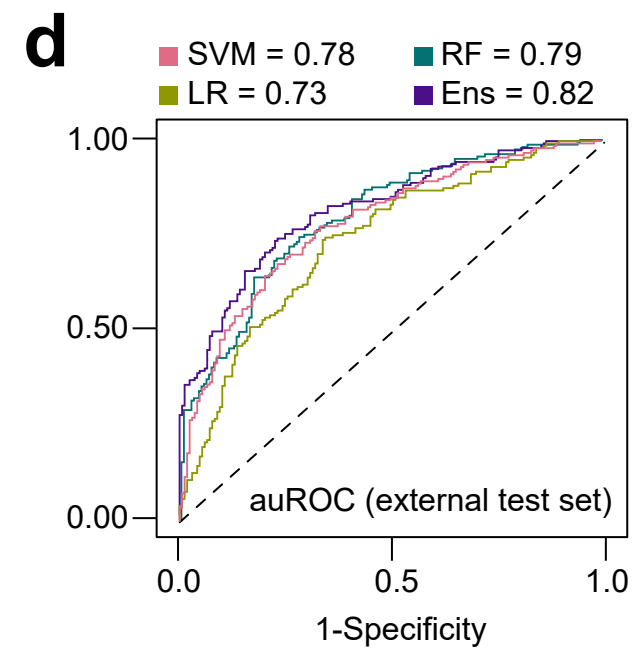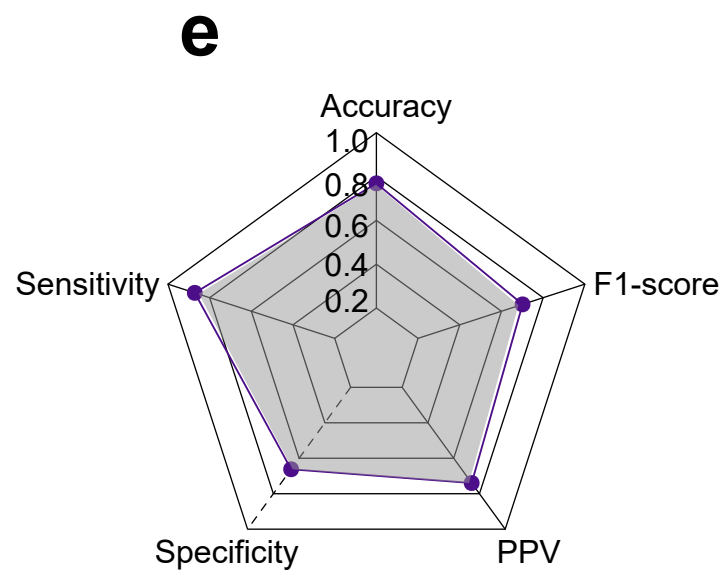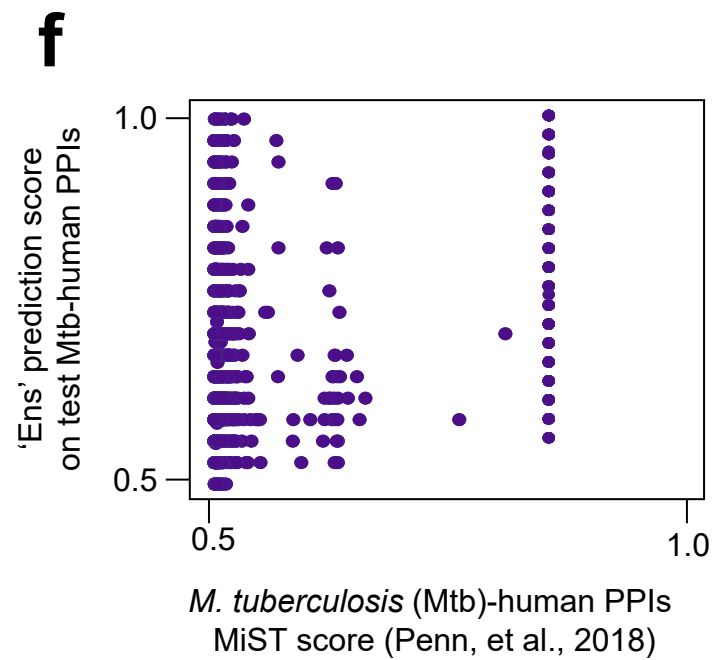

**Supplemental Figure 2**

Supplement: vbac038_Supplementary_Data [file vbac038_supplementary_data.zip › Supplementary_Figure2 .pdf]
